# Retrieving planktonic foraminifera from lithified rocks, examples from the Eocene limestones and marls (External Dinarides, Croatia)

**DOI:** 10.1016/j.mex.2023.102233

**Published:** 2023-05-26

**Authors:** Željko Ištuk, Štefica Kampić, Igor Felja, Matej Pavlović, Tamara Tudor, Ivan Jazvac, Đurđica Pezelj, Marija Horvat, Vlasta Ćosović

**Affiliations:** a Department of Geology, Faculty of Science, University of Zagreb, Horvatovac 102b, HR, 10000 Zagreb, Croatia; b Croatian Geological Survey, Sachsova 2, HR, 10000 Zagreb, Croatia; c Jazvaci 22, HR, 47201 Draganić, Croatia

**Keywords:** Planktonic foraminifera, Extraction, Limestone, Marls, Middle Eocene, Acetic acid, Croatia, *Extraction of foraminiferal tests from Eocene carbonates: application of the acetic acids and hydrogen peroxide methods*

## Abstract

Paleoecologic (paleoclimatologic) and biostratigraphic studies of pelagic and deep-water deposits rely on the identification of planktonic foraminifera. Here we report and compare the results of planktonic foraminiferal assemblages from the Middle Eocene indurated limestones and marls collected in the External Dinarides extracted with acetic acid of different concentrations (50%, 60%, 70% and 80%) and different reaction (exposure) times. The deposits originated within the Dinaric foreland basin, have been assigned to the so-called Transitional beds and Flysch, and are characterized by different ratio of carbonate content and degree of lithification. The aim of this paper is to compare the efficiency of the laboratory procedures for obtaining isolated specimens and to evaluate the impact of preparation procedure on the quality of tests (complete test vs. secondary dissolution effects). For each acetic concentration we assessed:

(1) the effectiveness of the treatment in terms of the time required for successful extraction of planktonic foraminifera, and
(2) the degree of dissolution by analyses of dissolution proxies, including the weight percentage of sieved residues after disaggregation and preservation features of the tests. Our results indicate that accurate taxonomic analysis of carbonate rocks requires the use of 60% acetic acid for a shorter reaction time, and hydrogen peroxide methods for marls.

## Graphical abstract

**Figure:**
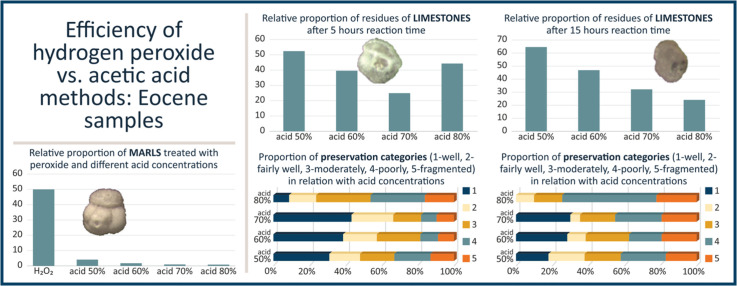

Specifications table

**Table:** 

Subject area:	Earth and Planetary Sciences
More specific subject area:	*Micropaleontology*
Name of your method:	*Extraction of foraminiferal tests from Eocene carbonates: application of the acetic acids and hydrogen peroxide methods*
Name and reference of original method:	*W. Wick, Aufbereitungsmethoden in der mikropaläontologie, Jahrebericht Naturhistorischen Gesellschaft, Hannover, 98 (1947) 35–41.*
Resource availability:	*Marls and limestones, Dinaric foreland basin.*

## Method details

**Fig. 1:**
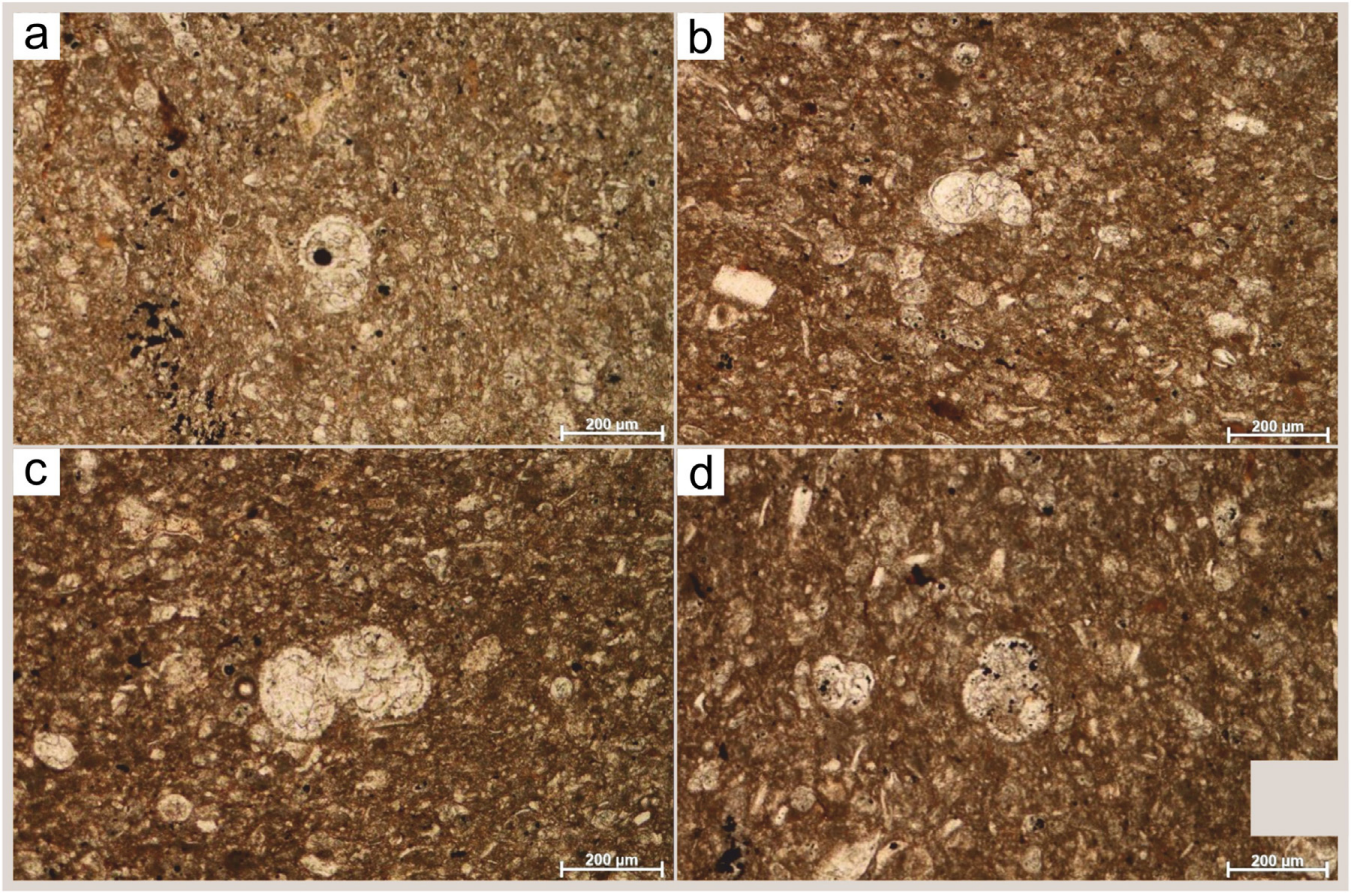
**(a-d)** Microphotographs of thiné sections of Transitional beds interpreted as calcilutite with planktonic foraminifera disperse in fine grained matrix (sample TG6). The planktonic assemblage (genera were identified only as *Acarinina* sp. (a) and *Subbotina* sp. (c), and indeterminable test sections b, and d) occupied 22.5% of the rock volume (point-counting method), while skeletal remains (due to their small size identification was impossible) constituted about 11.7% of the rock, with fine calcite grains making up the rest.

Two sets of carbonate samples of the Eocene age but different depositional settings were studied. One set of samples is from the Vinodol Valley (near Tribalj municipality, referred as TG6), from informal formation known as Transitional beds [1]. These beds are lithified limestones, originated in the upper part of the slope. The sediments are clastic biogenic carbonate rock dominant, calcilutite (Fig. 1). Planktonic foraminiferal tests constitute dominant biogenic allochems, were unevenly distributed in matrix composed of micritic size grains. Rare fragments of unidentified bivalves and smaller benthic foraminifera belonging to rotaliids, occur in the sediments. The chamber lumens of planktonic foraminifera (*Subbotina* sp., *Acarinina* sp., *Globigerinathek*a sp., *Turborotali*a sp., *Morozovella* sp.) were filled with matrix (Fig. 1c, combination of skeletal fine debris and micrite), or with sparitic infillings (recrystallized calcite, Figs. 1a, b, d). Diagenetic cement, also, appeared, as infillings of compaction fractures (Fig. 1). Based on identified larger benthic foraminifera species and genera (*Alveolina* sp.*, Asterocyclina* sp. and *Discocyclina* sp.) from thin sections, samples were assigned to Middle Lutetian [7]. The other set of samples is hemipelagic marls (Flysch deposits) from the island of Hvar (Podstine Bay). The sample (referred as PP4) with 73.44% CaCO_3_ [3] and is interpreted to be of Priabonian age [4].

**Fig. 2:**
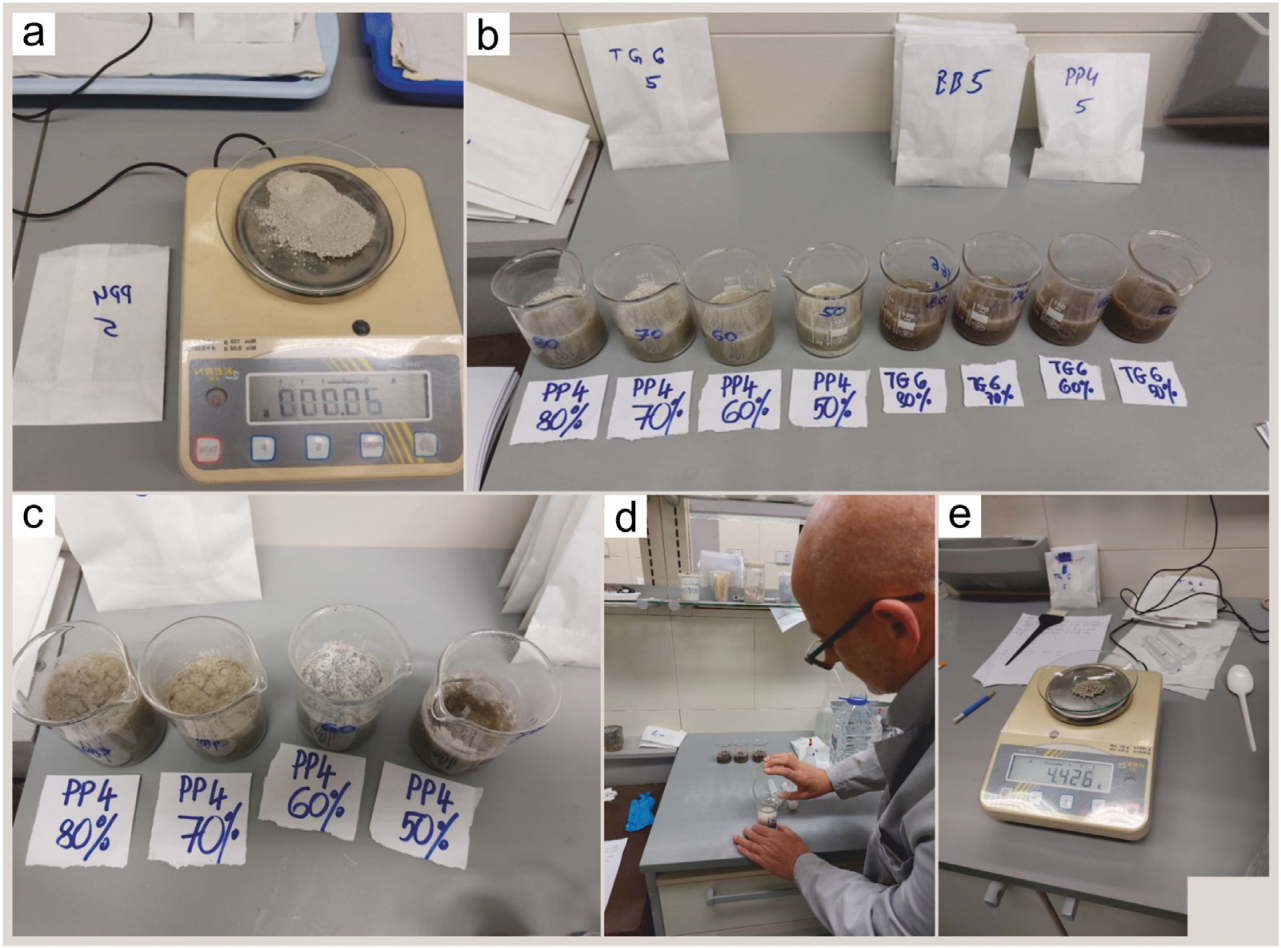
The main steps (a-e) in processing the samples with acetic acid of different concentrations: (a) crushed initial sample; (b) reaction of the solution of 50%-80% acetic acid and deionized water on the initial samples (PP4 and TG6); (c) reaction of acetic acid of different concentrations on the samples; (d) decantation after 15 hours of soaking and obtaining the residue; (e) weighing of samples > 1 mm separately after treatment with 80% acid concentration.

The procedure using mixture of acetic acid and deionized water (modified after Malik et al. [4]) consisted of the following steps (Fig. 2):

1. 100 g of limestones (2 times) and marls were broken into small fragments of max 5 mm in size (larger grains require more time to be disaggregated).
2. The crushed samples were divided into five subsamples (20 g per one subsample, [5]) and placed in glass beakers. Four subsamples were treated with a mixture of acetic acid and deionized water at different concentrations, whereas the fifth subsample was treated with a mixture of water and hydrogen peroxide.
3. Solutions of acetic acid (CH_3_COOH) of 50%, 60%, 70% and 80% mixed with 50%, 40%, 30% and 20% of deionized water were prepared. The level of the mixed solution was always kept about 2 cm above the sample level.
4. The subsamples were submerged in the solutions. The limestone and marl subsamples were left for 15 hours (limestones and marls), and in addition, one set of subsamples of limestones were exposed to solution for 5 hours. During the reaction an excess of calcium acetate crystals formed.
5. The washing procedure was the same in both methods. The disaggregated subsamples were washed thoroughly over a set of steel sieves (with mesh openings of 0.063, 0.125, 0.250, 0.500, and 1.00 mm) and dried at room temperature for the next 24 hours.
6. The residues from each sieve were weighted (Tables 1, 3) and transferred to labeled paper bags.

**Table 1:** Limestone (Transitional bed, TG6) sample: absolute and percentage weight of original sub-sample processed with H_2_O_2_ and with different concentrations of acetic acid exposed to reaction time of 5 hours and reaction time of 15 hours (in yellow) for each size fraction.
7. For evaluation, the dry sieving was performed over the 0.063 mm mesh, and approximately 100 randomly selected tests from each sample were picked (using microsplitter) and studied under the Olympus SZX7 stereomicroscope. We chose stereo microscopes because they are available in almost all laboratories where samples containing foraminifera are processed. The initial screening of treated samples always takes place under these instruments, and observations made would imply further steps for continuing laboratory work or collecting tests for further analysis. The scanning electron microscopy (SEM) imaging of selected tests is done for publications, because all studies are mainly carried in this way due to the time-consuming preparation of samples and the limited availability of SEM.
8. The quality of the sample residues was appraised by estimating the preservation status of the recovered foraminiferal tests. Fragmented, partially dissolved and undissolved specimens were described, counted, and ranked according to their state of preservation. The following preservation categories were assessed: well preserved (no clay-sized carbonate particles present on the test, original texture of wall mostly preserved, marked as #1), fairly well preserved (some clay-sized carbonate particles covered septal suture depressions, smooth, glassy test wall without “ornamentation”, #2), moderately preserved (test surface on spots were is not partially dissolved are being covered with clay-sized carbonate particle, #3), poorly preserved (tests were still in matrix with dissolution marks on test wall, #4), and fragmented (< half of the test; it must be considered that some tests may have been damaged during mechanical preparation of the samples, #5).

The stereoscopic images were taken by Olympus U-TV-1XC camera at the Department of Geology, Faculty of Science, University of Zagreb.

## Application of the method in the transitional beds limestones

### Hydrogen peroxide method

The traditional method proved to be unsuitable. The subsamples had the same granulometric properties as they had before soaked in a mixture of hydrogen peroxide and water.

### Acetic acid method

The samples of the Transitional beds showed different residue recoveries treated with different acid concentrations and with different duration of disaggregation processes. The weight of dry residues obtained with different mesh sieves, acid concentrations and reaction times are given in Table 1.

The 20 g of sample is reduced from 5.41 to 12.92 g when the sub-samples were soaked for 15 hours in a mixture of acetic acid of different concentrations and deionized water, and from 4.99 to 10.48 g when the disaggregation time lasted 5 hours (Table 1).

At acid concentrations of 50% to 80% left for 5 hours, the weight of the fraction(s) > 0.063 mm decreased with increasing acid concentration. The weight loss ranged from 47.6 to 75.05% of the total weight of 20 g, being lowest at an acid concentration of 50% and the highest at 70%. The weight of the finest fraction increased as a function of acid concentration (the weight of residue was highest when sub-sample was left in 50% acid concentration).

The steady weight loss with increasing acid concentration occurred when the sub-samples were exposed to dissolution for an extended period of time. The total weight loss ranged from 35.5% (sub-sample was left in 50% concentrated acid) to 72.5 % (weight loss in sub-samples treated with 80% acid). Interestingly, the weight of the finest fraction (> 0.063 mm) did not depend on the acid concentration (the highest weight at 60% and the lowest at 70% acid concentration).

**Fig. 3:**
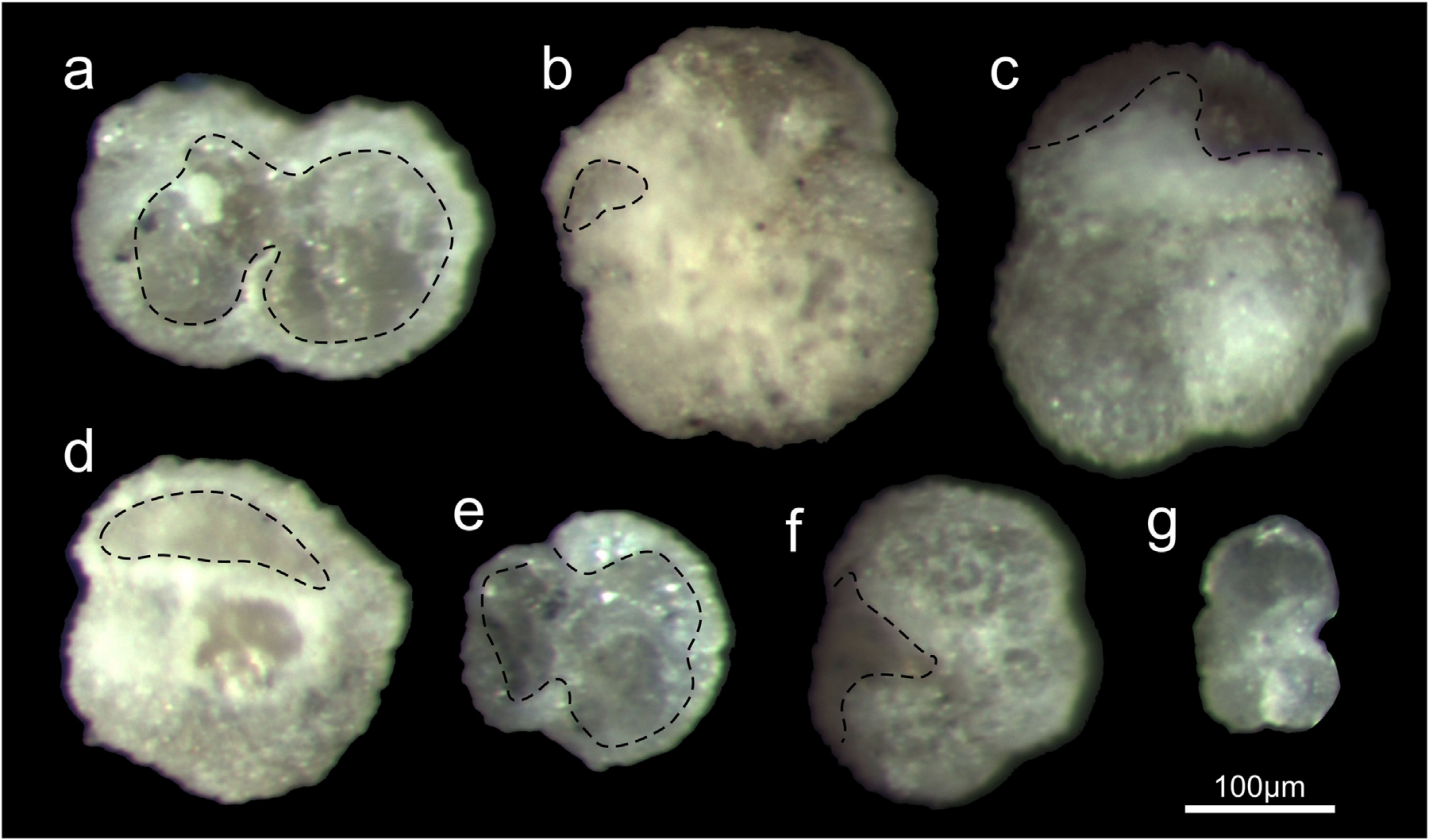
Examples of foraminiferal tests recovered from limestone (sample TG6) soaked in different concentrations of acetic acid for 5 hours (lines show boundary between preserved and dissolved wall), (a, g) planktonic tests (unidentified) recovered after treatment with 80% acid concentration (removed wall area cover several chambers, test fragmentation) (b) foraminiferal test (*Globigerinatheka* sp.) recovered after treatment with 60% acid concentration (partially dissolved wall of penultimate chamber); (c, d, e) planktonic tests (*Turborotalia* sp.,*Globigerinatheka* sp., unidentified) recovered from the residues after treatment with 70% acid concentration (variation of test conditions from partially dissolved wall to fragmented test with preserved original wall texture); (f) foraminiferal test (*Turborotalia* sp.) recovered after treatment with 50% acid concentration (wall of youngest chamber dissolved, walls of other chamber changed color, texture). These show the range of preservation categories according to acid concentration from well preserved, fairly well preserved, moderately preserved and partially dissolved.

The visual and binocular analysis of the residues revealed color change between rock and treated sub-samples. Foraminiferal tests were glassy opaque in appearance. The forms recovered from residue left after treating sub-sample with 80% acid were barren from original wall structure (internal molds for the most cases, Fig. 3g) and yet fine-grained material covered sutural depression. Still, generic (*Subbotina* sp., *Turborotalia* sp., *Globigerinatheka* sp., all cold-water genera [6]) to species identification was possible, depending on identification criteria for certain genera and species. The proportions between well and poorly preserved tests corresponded to the acid concentrations. The higher the acid concentration, the more partially to completely dissolved tests, fewer embedded tests, and a greater number of tests with exfoliated ultimate whorl were observed (Table 2; Fig. 3). Thus, the residue treated with 80% acid for 15 hours consisted of 10% fairly well preserved and 22.2% fragmented tests, while the residue treated with 60% acid for the same reaction time contained 28.4% well preserved and 19.3% fragmented tests.

**Table 2:** Absolute and relative (proportion) number of foraminiferal tests in preservation ranking categories of limestones, Transitional beds (TG6):#1: well preserved tests (wall mostly preserved intact or with only small amounts of matrix attached at septal suture; #2: fairly well preserved (wall surface covered with sporadically distributed etch spots and fine-grained particles masking septal suture); #3: moderately preserved (partially dissolved wall structure, septal suture mask with fine-grained particles); #4: poorly preserved (wall mostly dissolved, translucent tests); #5 broken tests (internal molds and fragmented).

Acid concentration	#1	#2	#3	#4	#5	#1	#2	#3	#4	#5
	Reaction time – 15 hours					Reaction time – 5 hours				
50%	17	19	19	24	16	29	16	18	19	12
60%	25	9	21	16	17	35	17	22	9	8
70%	28	5	18	24	17	45	24	16	9	10
80%		9	14	47	20	8	14	28	28	15


Acid concentration	#1%	#2%	#3%	#4%	#5%	#1%	#2%	#3%	#4%	#5%
	Reaction time – 15 hours					Reaction time – 5 hours				
50%	18	20	20	25	17	30.85	17.02	19.15	20.21	12.77
60%	28.4	10.2	24	18.1	19.3	38.46	18.68	24.17	9.89	8.79
70%	30.1	5.4	19.4	25.8	19.3	43.26	23.07	15.37	8.64	9.6
80%		10	15.6	52.2	22.2	8.6	15.05	30.1	30.1	16.13

Over 100 specimens were randomly picked from residues obtained from different concentrations of acid from limestones samples (TG6). A detailed analysis of the quality of the foraminiferal tests is shown in Table 2. The results after 5 hours of soaking are the following: (1) Most foraminiferal tests from the 50% concentration were well to fairly well preserved (etch marks covered limited surface areas), while tests without original wall textures, some parts covered with fine-grained material, and fragmented tests were common. (2) Well-preserved to partially dissolved wall textures characterized the majority of tests from the 60% concentration (81.4% of all tests belonged to preservation categories #1 to #3), and test surfaces were partially covered with fine-grained material. (3) A greater number of fragmented tests were found in the residues of the 70% concentration (9.6%), but the assemblages contained the greatest number of well- and fairly well preserved tests (66.4%). Some tests had partially disintegrated walls, and again, fine-grained materials covered some portions of the tests. (4) Most moderately to poorly preserved and fragmented tests were recovered after treatment with an 80% acid concentration and accounted for 76.3% of all tests recovered. (5) No differences in preservation of tests among representatives of thermocline foraminifera genera were observed.

With longer exposure time (15 hours) and higher concentration of acetic acid (80%), the percentage of internal molds and fragmented tests increased. The residues treated with an acid concentration between 50% and 70% for 15 hours had almost the same percentage of well-preserved tests (ranging from 38% at 50% to 35.4% at 70%), while after soaking in 80% acetic acid, not a single well preserved specimens was found, and 90% of all recovered tests were classified as moderately to poorly preserved. The comparison of the quality of the tests expressed as the sum of the well to moderately preserved tests extracted after 15 hours in a solution with an acid concentration of 50% - 70%, is classified as moderate, as the percentage of the three groups varied from 58% at 50%, to 62.6% at 60% and 54.9% at 70% acid concentration. The tests kept for 5 hours are considered quite good (as the percentages of such preserved tests range from 67% at 50% to 81.4% at 60% and 81.8% at 70%).

## Application of method in the marls

Four sub-samples of Podstine Bay sample (PP4) were treated with different concentrations of acetic acid over 5 hours, while the fifth subsample was soaked in H_2_O_2_ (30%) for 24 hours.

### Hydrogen peroxide method

**Fig. 4:**
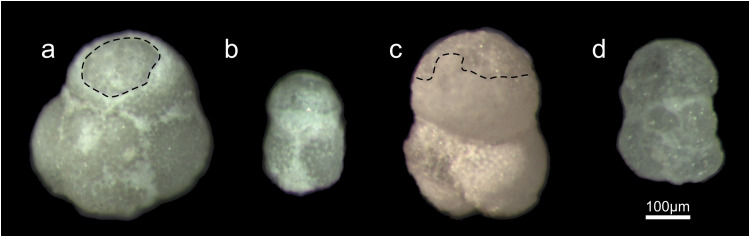
Examples of foraminiferal tests recovered from the marls (sample PP4) using different concentrations of acetic acid for 5 hours (the lines delineated the original wall texture from dissolved parts): (a) foraminiferal test (*Turborotalia* sp.) recovered after treatment with 50% acid concentration (partially dissolved test wall on the youngest chamber); (b) foraminiferal test recovered after treatment with 60% acid concentration (greater proportion of dissolved wall); (c) planktonic test (*Subbotina* sp.) recovered from the residue after treatment with 70% concentrated acid (the original wall was removed greatly); (d) planktonic test (*Subbotina* sp.) recovered after treatment with 80% acid concentration (the original wall was completely removed, part of the lumen infilling was, also, dissolved, translucent appearance). These show the range of preservation categories according to acid concentration from well preserved, fairly well preserved, moderately preserved and partially dissolved.

The percentage of the subsample broken up into particles between 0.5 and 0.063 mm was 2.5%. Planktonic foraminifera (representatives of genera *Subbotina* sp., *Turborotalia* sp. and *Globigerinatheka* sp.) have test surfaces partially covered with clay sized micrite, septal suture scars filled with fine-grained particles, and chamber lumens filled with sparitic calcite (recrystallized calcite; Fig. 4). Some tests showed damages like partially exfoliated (“peeled-off”) walls (Table 3).

**Table 3:** Marls (PP4) sample: absolute and percentage weight of original sub-sample process with H_2_O_2_ and with different concentration of acetic acid for each size fraction.

Sub-sample weight before treatment	Fraction >1 mm	Fraction > 0.500 mm	Fraction > 0.125 mm	Fraction > 0.063 mm	Total weight after treatment
20 g + H_2_O_2_	9.0 g	0.5 g	0.2 g	0.3 g	10.00 g
20 g + 50% acetic acid		0.01 g	0.38 g	0.43 g	0.82 g
20 g + 60% acetic acid			0.15 g	0.20 g	0.35 g
20 g + 70% acetic acid			0.09 g	0.10 g	0.19 g
20 g + 80% acetic acid			0.14 g	0.03 g	0.17 g


Sub-sample weight before treatment	Fraction >1 mm (%)	Fraction > 0.500 mm (%)	Fraction > 0.125 mm (%)	Fraction > 0.063 mm (%)	Total weight after treatment (%)
20 g + H_2_O_2_	45	2.5	1.0	1.5	50.0
20 g + 50% acetic acid		0.05	1.9	2.15	4.1
20 g + 60% acetic acid			0.75	1.0	1.75
20 g + 70% acetic acid			0.45	0.5	0.95
20 g + 80% acetic acid			0.7	0.15	0.85

### Acetic acid method

Acetic acid treatment resulted in consistently low residue weights regardless of concentration. Weight loss varied from 99.15% in mixture of 80% of acetic acid and deionized water to 95.9% in solution of 50% acetic acid concentration with water. The largest weight loss of the fraction > 0.063 mm may be due to a high dissolution rate resulting from the crushing of the samples into the fine-grained homogeneous size fraction and the resulting increase in the contact area with the solution [5,7]. The foraminiferal tests (*Subbotina* sp., *Turbototalia* sp.) were milky opaque white and well preserved (original wall texture, only apertures were covered with clay-sized particles) in the case of treatment with 50% and 60% acid concentration. The tests obtained from 70% and 80% acid concentrations showed partially preserved original wall structure (Fig. 4), tests were often broken and, original wall dissolved and tests opaque, grayish in color.

## Method validation

The Paleogene sediments of the External Dinarides, exposed along the eastern Adriatic coast, were deposited in the Dinaric foreland basin, in the depositional environments ranging from shallow - water carbonate ramps, to transitional settings between shallow- and deep-water basins [8,9]. They contain diverse assemblages of foraminifera, whose composition, abundance, and diversity depend on the depositional settings. Shallow-water carbonates are rich in larger benthic foraminifera, while hemipelagic and pelagic deposits (indurated limestones and marls, mudrocks with different calcite content [3], contain predominantly planktonic and smaller benthic foraminifera. Foraminifera are protists that for decades were used as biostratigraphic or paleoenvironmental indicators (i.e., depth, temperature, salinity, oxygen contents…). Their application relies on their tests that contain many important information for species identification, preservation conditions (like dissolution marks, fragmentation) essential for their use for isotope analysis and abundances. Species identification criteria for larger benthic foraminifera differs from those for smaller benthic and planktonic foraminifera. A necessary step to use large benthic foraminifera for species identification is the preparation of oriented test sections (making thin sections) or acetate peels for revealing inner structural elements, whereas the species identification of smaller benthic and planktonic foraminifera is based on studying of external morphological characteristics in isolated specimens. Thus, if heavily lithified limestones or marls contain smaller benthic or planktonic foraminifera, specimens must be isolated from the matrix. The use of fossil assemblages of planktonic foraminifera for paleoceanographic reconstruction is based on a quantitative count. The question arises, how to get standard aliquots from lithified samples? Various methods have been used to transform the bulk sediment sample into usable material. The commonly used laboratory methods for disaggregation of marls and marly limestones, dating back to the 1940s [10], are dissolving the sample (previously physically crushed into fragments of maximum 5 mm) in hydrogen peroxide (H_2_O_2_) and washing soda (sodium carbonate, Na_2_CO_3_).

The cold acetic acid method has been widely used to extract microfossils from indurated rocks with more or less success [[11], [12], [13], [14], and references therein]. Many studies have tested different concentrations of acetic acid and reaction times (from 6 hours to 14 days) on different sample sizes, different lithological characteristics, and age attribution [[4], [5],[11], [12], [13], [14], [15], [16], [17], [18], [19], [20]]. The free hydrogen ions in the acid attack the rock matrix, dissolving it and breaking it apart [7]. Working with fine grained carbonate rocks is particularly challenging because of the high rate of dissolution. For reliable paleoecological interpretation sufficient foraminiferal tests are needed (standard: 200–300 tests per sample) and taxonomic identification requires tests with visible fine structural elements.

The aim of this study was to propose the most suitable technique for the extraction of foraminiferal tests in sufficient quantity from lithified limestones (fine grained) and carbonate rich marls. In order to get the results. samples of different lithology were treated with hydrogen peroxide and acetic acid and the quality and amount of skeletal remains were analyzed. The effectiveness of retrieving microfossils from sediments depends on the mineralogy of the sediment matrix, grain size, and degree of lithification. We compared (1) the time required to recover extracted foraminiferal tests and, their preservation conditions; and (2) conducted a comprehensive evaluation of acid residue recovery following the procedure of Malik et al. [4] in terms of test preservation, specimen cleanliness and fragmentation, and degree of dissolution. Two types of samples were analyzed, the Middle Eocene lithified limestones, belonging to the informal formation known as Transitional beds [2,21] and marls (with a CaCO_3_ content of 73.44%) deposited in the basin during the Priabonian [3,22].

We conducted experiments with chemical treatments to disaggregate marls (mudstones) and indurated limestones, which differently affected the foraminiferal tests. The chemical agents used in the solution were hydrogen peroxide and acetic acid in four different concentrations (50%-80%) and different reaction times (5 and 15 hours). Although mineral calcite reacts with acetic acid, fossil remains can “resist” to be eliminated. Costa de Moura et al. [12] suggested the impurities present in the matrix of carbonate rocks provide boundaries for the acid to work on more effectively, whereas the pure biogenic carbonate of fossil test can be considered more impermeable to acid.

We have found that the hydrogen peroxide method was effective in preparing planktonic foraminifera (Fig. 4) for their further micropaleontological analysis from marls (even if they have a calcite content of 73.44% %). This method allowed the extraction of a large number of foraminiferal tests necessary for paleoenvironmental interpretation (about 300 specimens Table 3). In the present experiments, the marl sample was crushed into small fragments, weight and soaked into the mixture of 30% peroxide and water for 24 hours. This method combines effects of chemical dissolution of organic components and mechanical dissection through CO_2_ pressure in pores of rock sample by reaction with H_2_O_2_.The size of the particles was important because the smaller they were, the more effectively the hydrogen ions could dissolve and break up the matrix. Planktonic foraminiferal tests obtained from marls were diagenetically altered by recrystallization of the chamber lumen, masked aperture, and not clean with sediment matrix attached at them. No additional solution-induced damage was detected, confirming that the method provided good results.

For the recovered tests from lithified samples five points were discussed: the duration of the process, the solution concentration, the amount of residue, and the cost-effectiveness (conditions of the recovered tests), as well as the relation between the degree of preservation and genus affiliation (Tables 4,5). Preservation conditions of foraminiferal tests from marls, treated with cold acetolysis of different concentrations were different, ranging (Table 2; Fig. 3) from well preserved, partially dissolved to fragmented. Our experiments showed that amount and quality of foraminiferal tests “liberated” from lithified rocks depended on the acid concentration and reaction time. After 15 hours of soaking in different acid concentrations (Table 2), the greatest amount of well preserved and fairly well preserved test was found in the residues treated with 50% and 60% concentrations, while the proportion of better preserved tests decreased linearly with increasing concentration. The increase in acid concentration was positively correlated with the degree of damage to wall structures, the abundances of partially dissolved tests in > 0.063 mm fraction, and the greater abundances of translucent tests. As the acid concentration increased, the abundance of the liberated tests increased when the reaction time was 5 hours. Thus, residue obtained after treatment with 50% acid was characterized by a rare occurrence of foraminifera in the finest fraction, many tests were still attached to the matrix. The test surfaces were barren of ornamentation (muricae, pustules, cancellate surface), and the depression of the septal sutures were always covered (regardless of the concentration of acid) with clay-sized micrites (Fig. 3, Table 2). Among the representatives of the planktonic genera, there were no differences between the percentages of well and poorly preserved specimens. We suspect that this is related to their test morphology and biomineralization processes, since they all belong to deep dwellers [8] and thus are adapted to cold water conditions. The foraminifera that lived in the same water zone below the mixed layer had the same wall chemistry, which seemed to be purer than that of the matrix (defined as skeletal hash particles, silt particles of different origin) and more resistant to the corrosive effects of the solution [7]. Most well preserved and fairly well-preserved foraminiferal tests were retrieved from the residues after treatment with 60% and 70% acid concentrations. Although the overall weight of residues > 0.125 mm remaining after reaction with 80% acid was considerably reduced compared to the weight of residues of the same size treated with a lower concentration acid, the tests were still numerous, but many tests were fully and partially dissolved, and all studied tests had covered septal sutures (Fig. 3, Table 2). It must be emphasized that the most studies of planktonic foraminifera were from 0.063 and 0.125 mm fractions [23], so the amount of residue from these fractions plays an important role in considering the best methods. The difference between 60% and 70% acid concentrations over a processing time of 5 hours was the greater proportions of partially dissolved tests completely removed from the matrix and abundances of tests with “barren” surfaces (erased original wall structure, etched and corrosion signs) in the residues treated with 70% acid concentration. Considering the quality and number of tests (preserved criteria necessary for species identification) and the amount of residues, the 60% concentration of acid gave the most promising result, as suggested by Malik et al. [4]. The application of acetic acid was destructive and can alter the composition of foraminiferal assemblages by reducing the number of some taxa that are more susceptible to dissolution. The relationship between preservation categories and specific genera was analyzed only for the lithified samples treated with different acid concentrations for 5 hours (Tables 4, 5). Acetolysis does not appear to be recommended for calcite-rich marls because very few residues remain after treatment, while a 15-hour treatment of the lithified samples resulted in a significant reduction of well-preserved or fairly well-preserved tests. The analysis showed that the thermocline forms (*Subbotina* sp., *Turborotalia* sp., *Globigerinatheka* sp.) apart from being dominant were generally better preserved. The frequency of well-preserved subbotinids varied from 5.37% at 80% to 27.47% at 60%, and the same trend was found for turborotaliids and globigerinathekas. Their high abundance is the result of the dominance of this group of foraminifera during the sedimentation [24], when the inhabitants of the mixed layers (*Acarinina* sp., *Morozovella* sp.,) were less abundant. In addition, it is known that foraminifera that are surface dwellers are much more susceptible to dissolution, which may also be the reason why acarininids and morozovellas were present in very low numbers [25].

**Table 4:** Absolute number and relative proportion of the recovered genera from the TG6 sample, > 0.063 mm fraction at processing time of 5 hours at different acid concentrations.

Concentration of acid	*Acarinina* sp.	*Morozovella* sp.	*Subbotina* sp.	*Turborotalia* sp.	*Globigerinatheka* sp.
50%	6	5	31	27	25
60%	1	1	41	22	26
70%	2	1	38	32	31
80%			44	26	23


Concentration of acid	*Acarinina* sp.	*Morozovella* sp.	*Subbotina* sp.	*Turborotalia* sp.	*Globigerinatheka* sp.
50%	6.38%	5.31%	32.98%	29.78%	26.59%
60%	1.1%	1.1%	45.05%	24.17%	28.57%
70%	1.92%	0.96%	36.53%	31.73%	29.8%
80%			47.31%	27.95%	24.73%

**Table 5:** Absolute number and relative proportion of five genera of planktonic foraminifera in preservation ranking categories of limestones, Transitional beds (TG6) after 5 hours of soaking in different acid concentrations.

Legend: #1 - well preserved tests (wall mostly preserved intact or with only small amounts of matrix attached at septal suture; #2 - fairly well preserved (wall surface covered with sporadically distributed etch spots and fine-grained particles masking septal suture); #3 - moderately preserved (partially dissolved wall structure, septal suture mask with fine-grained particles); #4 - poorly preserved (wall mostly dissolved, translucent tests); #5 - broken tests (internal molds and fragmented). The heat diagram shows the proportion up to 5% in light gray, from 5.1% to 10% in medium gray, and above 10.1% in dark gray.

## Conclusion

The main objective of this study was to improve, validate and apply the most optimal methods in liberating planktonic foraminiferal tests from indurated carbonate and mudrocks. To do this we focused on two disaggregation techniques on pelagic marls and indurated hemipelagic limestones. With respect to the quality of the recovered foraminiferal tests the following recommendation stands out:

1. The chemical treatment of marls with a calcite content of 73.44% with hydrogen peroxide allowed extraction of a significantly high number of foraminiferal tests, thus proved to be suitable for recovering planktonic foraminifera (limited evidence of etching, nor signs of fragmentation of tests and preserved test surface in accordance with their preservation conditions).
2. The acetic acid method gave promising results in extracting tests of planktonic foraminifera from strongly indurated limestones exposed to acetic acid concentrations of 50%-70% for 5 hours.
3. The amount of partially dissolved (a large portion of the etched and corroded surface of the tests) or dissolved foraminiferal tests recovered from the lower (50%) concentration of acid was lower than from the higher (80%) concentration, regardless of whether the samples were treated for 5 or 15 hours.
4. A longer processing time (15 hours) resulted in an overall poorer preservation quality of the extracted tests (greater proportion of poorly preserved, fragmented, and translucent tests).
5. The efficiency of the acetic acid procedure in extracting planktonic foraminifera made most advisable the use of a mixture of 60% acid and deionized water and 5 hours of exposure time since numerous tests with partially original wall structure were preserved.

## CrediT author statement

**Željko Ištuk:** Conceptualization, Investigation, Methodology, Writing - original draft. **Štefica Kampić**: Methodology, Investigation. **Igor Felja**: Investigation, Validity tests, Writing - original draf. **Matej Pavlović**: Methodology, Investigation. **Tamara Tudor**: Methodology. **Ivan Jazvac:** Visualization, Methodology. **Đurđica Pezelj**: Visualization; Project administration. **Marija Horvat:** Methodology, Preparation and editing draft. **Vlasta Ćosović**: Conceptualization, Methodology**:** Writing - original draft, Preparation, Editing.

## Declaration of Competing Interest

The authors declare that they have no known competing financial interests or personal relationships that could have appeared to influence the work reported in this paper.

## Data Availability

Data will be made available on request.
